# Experiences of self-harm, suicidal ideation and mental health care among autistic youth

**DOI:** 10.1177/13623613251366863

**Published:** 2025-09-01

**Authors:** Amanda Sabo, Jo Robinson, India Bellairs-Walsh, Linda Taimre, William Nguyen, Lisa Phillips, Michelle Lamblin, Eleanor Bailey

**Affiliations:** 1 The University of Melbourne, Australia; 2 Orygen, Australia

**Keywords:** autism, help-seeking, mental health care, self-harm, suicidal ideation, youth

## Abstract

Autistic youth experience high rates of self-harm and suicidal ideation, and substantial barriers to adequate mental health care. Research suggests that mental health services may not meet the needs of autistic individuals, though autistic youth experiencing self-harm and suicidal ideation are underrepresented in this literature. The present study aimed to explore experiences of self-harm, suicidal ideation and mental health care among a sample of autistic youth recruited through two Australian government-funded youth mental health services. Online interviews with seven autistic participants aged 15–23 years were transcribed and thematically analysed using a reflexive approach. Four themes and two subthemes were generated: (1) social rejection as risk, and connection as protection; (2) overwhelming emotions can lead to self-harm and hinder help-seeking; (3) feeling (mis)understood and (in)adequately accommodated by clinicians (comprising two subthemes related to poor understanding and accommodation of autism within mental health services); and (4) safety planning can feel like a box-ticking exercise. These findings highlight the importance of social connectedness as a protective factor against self-harm and suicidal ideation for autistic youth, and the necessity of improving understanding and accommodation of autism in mental health care settings and suicide interventions.

**Lay abstract:**

Autistic people under the age of 25 experience high rates of self-harm and suicidal thoughts. Previous research has found that mental health care provided by professionals like psychologists might not meet the needs of autistic people. However, this research has usually focused on autistic adults, so less is known about the experiences of younger autistic people. In this study, we spoke with seven autistic young people aged between 15 and 23 years, and asked about their experiences of self-harm, suicidal thoughts and mental health care. Many participants had experienced social rejection or bullying, which contributed to their self-harm and suicidal thoughts. However, positive relationships with family, friends and others were a source of support when they were struggling. Self-harm was described as a way to cope with strong negative emotions, but many participants found it hard to talk about or describe those emotions, which made it difficult to get support. The help they received from psychologists for their self-harm and suicidal thoughts was impacted by how well the psychologist understood autism, and whether they were willing to accommodate the participants’ individual needs and preferences. Participants had created suicide safety plans as part of the mental health care they received, but many felt like they had to do this just for the sake of doing it, rather than creating a plan that was truly helpful.

Autistic youth under the age of 25 are at least twice as likely to die by suicide than their non-autistic peers (Kirby et al., 2019; O’Halloran et al., 2022). Self-harm and suicidal ideation, two prominent suicide risk factors, are common in autistic youth: as many as one in three report lifetime self-harm and one in four report lifetime suicidal ideation (O’Halloran et al., 2022), with similar rates of suicidal thoughts and behaviours reported by autistic males and females across the lifespan (Moseley et al., 2024; Schwartzman et al., 2025). These high rates of self-harm and suicidal ideation are likely influenced by a range of individual and systemic factors. For instance, autistic youth report high prevalence of certain mental health conditions (e.g. depression and anxiety; Hollocks et al., 2023), bullying victimisation, adverse childhood experiences (Song et al., 2025), and loneliness (Deckers et al., 2017); all of which are risk factors for self-harm, suicidal ideation and suicide among this population (O’Halloran et al., 2022). Furthermore, autistic people experience unique barriers to receiving adequate mental health care, such as poor availability of autism-specific services and inadequate accommodations (Crane et al., 2019; Gilmore et al., 2022), in addition to common barriers experienced by non-autistic consumers (e.g. long wait times and high costs).

Mental health services represent an important avenue for detection and prevention of self-harm and suicide risk. However, generic services are often reported as failing to meet the mental health care needs of autistic youth (Adams & Young, 2021; Delgado et al., 2024), which can contribute to premature withdrawal from care (Crane et al., 2019). In addition, autistic adults receiving mental health care for self-harm and suicidal ideation have reported that poor provider knowledge of autism and a lack of appropriate treatment options contributed to worsening feelings of hopelessness and isolation (Camm-Crosbie et al., 2019). However, the experiences of autistic youth seeking mental health care for self-harm and suicidal ideation are underexplored in the current literature. To address this, and to identify opportunities to improve the quality of care provided to this population, the present study sought to explore experiences of self-harm, suicidal ideation and mental health care received for self-harm or suicidal ideation among autistic youth.

## Methods

This study received approval from the Melbourne Health Human Research Ethics Committee (HREC 2020.166).

### Research orientation and reflexivity

The materials and analytic approach utilised in this study are aligned with a constructivist position, which assumes that meaning and knowing is informed by an individual’s perceptions, context, prior experiences and subjectivity (Creswell & Poth, 2016). Utilising a qualitative methodology informed by this theoretical orientation enabled us to examine, in depth, how youth with a certain set of lived experiences and characteristics perceived and made sense of their experiences related to the study aims.

The research team comprised six suicide prevention researchers (E.B., J.R., I.B.W., L.P., M.L. and A.S.; of whom, two were also psychologists: E.B. and L.P.), as well as two postgraduate psychology students (L.T. and W.N.). Of the researchers involved in data collection (E.B., I.B.W., L.T. and W.N.), most were White, cisgender and not autistic, with varying degrees of professional experience working with autistic people and/or youth with lived experience of self-harm and suicide, in research or mental health care settings.

Of the researchers involved in data analysis (E.B. and A.S.), E.B. is a White, cisgender, non-autistic woman who works clinically with autistic youth experiencing suicide risk and leads research into youth suicide prevention in clinical settings and across the Australian population. A.S. is a White, cisgender, autistic woman who is currently a graduate researcher in suicide prevention, and works as a research assistant supporting the development, implementation and evaluation of youth suicide prevention interventions. Both have prior experience with qualitative research and thematic analysis.

### Participant recruitment

Purposive recruitment was conducted through two government-funded youth mental health services in Australia: Orygen Specialist Program (OSP) and headspace. OSP and the five headspace centres that we recruited through specifically service youth living in metropolitan Melbourne. This approach enabled us to reach individuals with the target experiences and characteristics, and strengthened risk management protocols by ensuring that participants were receiving mental health support from their clinicians at the time of participation.

Two members of the research team (E.B. and I.B.W.) provided study information to clinicians at recruitment sites across two recruitment periods (February to July 2021; and May to December 2022). Young people who were determined by their treating clinician to likely meet the study’s eligibility criteria were referred to the study. Individuals were eligible to participate if they were aged between 15 and 25 years; had a formal autism diagnosis or suspected/self-identified as autistic; had experience of seeking help from OSP or headspace for suicidal ideation and/or self-harm; and were fluent in English. In addition, participants had to be regularly attending scheduled appointments with their treating clinician, willing to nominate an emergency contact and able to provide informed consent. Those who were deemed by their clinician to be at high risk of suicide at the time of recruitment were excluded.

E.B. and I.B.W. contacted eligible individuals to provide a plain language statement, answer any questions regarding participation or consent, and confirm their eligibility. Participants were offered accommodations such as providing written responses to interview questions instead of spoken responses, bringing a support person to the interview, taking breaks during the interview, or splitting the interview across two or more sessions. Informed consent was then obtained from all participants, as well as parental consent for those under the age of 18, via an online consent form. Participants were provided with the interview schedule in advance of their chosen interview date.

Sixteen prospective participants were referred to the study, of whom, seven provided consent and participated in interviews. Of those who did not participate, five declined (four young people and one parent of a young person declined consent), three were discharged from the service, and one agreed to participate but did not return the consent form.

Of the final sample (*n* = 7, aged 15–23 years), three participants self-described their gender as female or girl, and four participants used various other terms to self-describe their gender identity (two identified as nonbinary, one identified as transsexual and one as trans female). All participants were completing or had completed a high school education up to year 10 or above, and three participants were undertaking vocational education. Self-reported age of autism diagnosis varied from 3 to 20 years, with most diagnosed between the ages of 11 and 16 years. One participant was awaiting formal diagnosis. All participants resided in Victoria, Australia. Data on demographic characteristics such as race/ethnicity and socioeconomic status were not collected.

### Data collection

Each participant attended a semi-structured interview, conducted using Zoom video conferencing software, between March 2021 and October 2022. Interviews were facilitated by at least one member of the research team (E.B., I.B.W., L.T. and W.N.); those that were facilitated by the postgraduate students (L.T. and W.N.) were also attended by a more senior researcher (E.B.). Interviews were guided by a semi-structured interview schedule, which included questions relating to experiences of self-harm and/or suicidal ideation, experiences of seeking help for self-harm and/or suicidal ideation, and experiences of safety planning. The full interview schedule is provided in Supplemental Appendix 1. Additional topics addressed in the interview schedule were the focus of Master’s theses prepared by L.T. and W.N., and are not discussed here. Interviews ranged from approximately 30–90 min, and participants were reimbursed $30AUD per hour for their time. All interviews were audio recorded and transcribed for analysis.

### Data analysis

Reflexive thematic analysis was the chosen analytic approach, as it enabled the generation and interpretation of themes exploring shared meaning across diverse experiences (Braun & Clarke, 2022). In analysing and reporting on this data, we (E.B. and A.S.) engaged in introspective reflection to develop self-awareness of our own perceptions, experiences and subjectivity related to the research topics (Finlay, 2002). Notably, we reflected on how our perceptions were shaped by our experiences as a mental health clinician (E.B.) and autistic person (A.S.), respectively.

Analysis was informed by Braun and Clarke’s principles for reflexive thematic analysis (Braun & Clarke, 2019). One researcher (E.B.) reviewed and coded interview transcripts in NVivo 14 (Lumivero, 2023). Two researchers (E.B. and A.S.) then generated initial themes by mapping coding output and sorting codes based on shared meaning; this was undertaken independently, enabling us to navigate our own subjectivity and interpretations of the data. We then met to refine the themes together, which involved collapsing overlapping ideas, and discussing theme names and definitions. We explored areas of overlap and disagreement to understand the nuance of each of our approaches, and to deepen our interpretation. Theme names, descriptions and decisions regarding illustrative quotes were further refined with feedback from a researcher who had been involved in data collection, but not preliminary analysis (I.B.W.).

## Results

Through analysing data related to participants’ experiences with self-harm, suicidal ideation and mental health care received for self-harm or suicidal ideation, we generated four themes and two subthemes, which are reported below.

### Theme 1. Social rejection as risk, and connection as protection

Experiences of social isolation or rejection were common, and appeared to be detrimental to participants’ self-perception. They were discussed as direct triggers for suicidal ideation or self-harm:

I had no friends, I was always being bullied. The thoughts would just be that my life sucks. (P7, discussing thoughts experienced when they were suicidal)

If I perceive any kind of rejection, I punish myself for doing so. Because I must have done something wrong. (P2, discussing self-harm triggers)

A common idea underpinning participants’ experiences with interpersonal relationships was a sense of difficulty forming close connections with others, and the negative impacts of this on their wellbeing: ‘I had no friends. It was very depressing’ (P7). For some, a sense of isolation was influenced by feeling ‘different from the others’ (P4). One participant described how growing up as the only autistic person in their family ‘made it hard to feel accepted’ (P1) and that being one of few autistic people in their school community made them a target for bullying:

Growing up in a main-stream school, you didn’t really see a lot of spectrum kids unfortunately. Unlike now. So, it was something that you get bullied for. (P1)

In some cases, experiences of bullying motivated masking behaviour (efforts to conceal autistic traits or suppress emotional expression), which was described as a ‘reflex’ to ‘pretend to be normal’ (P2) out of fear of further judgement or mistreatment.

Conversely, connection to others appeared to be an important protective factor that supported participants’ wellbeing and helped them to cope with distress or urges to self-harm. Most participants identified a specific family member (e.g. a parent, grandparent, sibling or child) as a reason that they had delayed acting on thoughts of self-harm or suicide in the past: ‘I don’t want to hurt my family. So that’s sort of stopped me’ (P4).

One participant stated that they would ‘immediately seek out friends’ (P2) as a source of support whenever they felt unsafe because of their thoughts of self-harm or suicide. Another echoed this sentiment by discussing how having supportive friends and teachers who knew about their self-harm was helpful during times when they were struggling:

I had my best friend help, who knew [about my self-harm]. She helped as much as she could. So, I had a support group of people who knew and could help. I had some support teachers that knew. (P1)

### Theme 2. Overwhelming emotions can lead to self-harm and hinder help-seeking

Difficulty tolerating strong emotions was often discussed as a precursor to self-harm. Some described self-harm as ‘an emotional release’ (P1) for anger or anxiety, or a way ‘to cope with strong emotions because I don’t really know how to manage them because I feel things really intensely’ (P3).

In some cases, self-harm appeared to be a way of feeling physically grounded in response to these intense thoughts and emotions. During experiences of heightened emotional overwhelm or when having thoughts of suicide, these participants used self-harm to create physical feelings to distract from their internal state:

When I self-harm, I’m generally pretty overwhelmed and just need somewhere to put my energy, need something to distract me . . . self-harm is almost like a way to bring me back, to just feel something physically, ground me a little bit. Sometimes, that’s helpful for my suicidal thoughts, because it just brings me back into the moment, and I’m not stuck in my head. (P6)

. . . I needed something to make me feel. So, I’d self-harm because there was just too much going on in my head that I was going to explode. Then when I was having my suicidal thoughts, I was done. I was done with the feeling, I was done with the volcanoes in me. (P1)

Experiencing prolonged overwhelming emotions made it difficult for some participants to recognise when they were reaching the point of crisis. This meant self-harm could occur without ‘always think[ing] about it’ (P6) and that ‘sometimes I didn’t even know I’d done it, until afterwards’ (P1).

In these situations, difficulties identifying or communicating about strong feelings before the point of overwhelm appeared to impede timely help-seeking and support. For some, this was exacerbated by reluctance to express or talk about emotions around others in general, with common experiences of concealing emotions day-to-day: ‘I don’t show emotion so I’m really good at hiding them’ (P7), ‘I know from experience that I hide my true emotional state from everyone’ (P2); including in mental health care settings: ‘I don’t really like talking about my feelings and it just never helped’ (P5).

In addition, one participant described their inability to communicate verbally during periods of heightened distress, which further limited their ability to seek formal or informal help: ‘. . . when I’m in crisis, I go nonverbal. I can’t call anyone’ (P6).

### Theme 3. Feeling (mis)understood and (in)adequately accommodated by clinicians

Participants’ experiences receiving mental health care for self-harm or suicidal ideation were directly impacted by the degree to which their clinician was perceived to be knowledgeable about autism (subtheme 1), and willing to accommodate their sensory and communication needs (subtheme 2). Better experiences of care consistently appeared to stem from feeling understood and accommodated as an autistic person, while perceptions that the clinician had poor knowledge of autism appeared to result in worse experiences of care and poorer recognition of sensory and communication needs.

#### Subtheme 1. Carrying the burden of education and advocacy

Learning more about autism, including through receiving an autism diagnosis, helped some participants ‘understand how I work and how I function with things’ (P4) and enabled them to ‘advocate for myself a lot better’ (P6) in mental health care settings. However, the self-understanding that some participants felt they gained through their lived experience and autism diagnosis was contrasted by experiences where they felt their treating clinician did not believe they were autistic, or did not take the time to understand them as an autistic person. These experiences contributed to adverse outcomes including worsening distress, being ‘pretty reluctant to ask for help’ (P6) in future, or feeling that the care they received was not suitable for their specific needs and capabilities:

When I say I’m autistic, people don’t really believe me, and I get things like, ‘Ah, like are you sure? But you don’t look autistic’, and so because people don’t view me as autistic as well, they’re not willing to accommodate my support needs. (P6, discussing interactions with mental health clinicians)

It’s hard [. . .] to be able to work with a clinician who’s meant to help you, when they’re not working to get to know what helps you. What you can and can’t cope with. (P1)

Furthermore, participants who perceived their clinicians as having poor knowledge of autism described having to spend additional time and effort providing education and correcting misinterpretations of their behaviour during sessions:

I feel like a lot of the time, I’m doing a lot of the work to educate clinicians, when it should be the other way around. (P6)

I’ve had to explain that no, I’m not distracted. Me looking out the window is actually me focussing on you talking. (P2)

Conversely, participants who felt their clinicians had better knowledge of autism or greater willingness to understand their individual needs described better experiences of care, including feeling ‘significantly more productive’ (P2) during sessions, and being able to ‘focus on other things, like getting the help that I need, rather than me educating them . . .’. (P6). Feeling understood and supported by a knowledgeable clinician was perceived as beneficial to the therapeutic relationship and effectiveness of care:

. . . the reason why I found her really helpful, is just because she’s educated on autism, and she’s autistic herself . . . I feel like her knowledge on autism is so helpful, because it’s like she understands me a lot better. (P6)

#### Subtheme 2. Accommodations enable better experiences of care

Instances where participants perceived their clinicians as lacking knowledge about autism went hand-in-hand with perceptions that mental health services were ‘really lacking in their knowledge and awareness’ (P6) of autistic consumers’ sensory and communication needs. This included sensory factors within the physical environment, such as lighting: ‘It’s not just, I don’t like bright lights, it’s a specific type of light that I don’t like’ (P2), as well as sensory considerations related to specific strategies such as breathing exercises:

. . . she recommended to me breathing exercises for my anxiety, but the problem is . . . I am incredibly touchy about my breath. If the air is warm and or humid, I can sometimes be–hyperventilate and it can–and it causes a lot of stress in me. (P2)

Participants who spoke about particularly positive experiences of care described clinicians who had been willing to understand and accommodate their sensory and communication needs. Examples of accommodations made by clinicians included providing fidget toys to support focus or approaching conversations slowly and discussing one topic at a time. One participant felt better able to engage during sessions when talking with a clinician who was accepting of their conversation style. Based on their description of this interaction, the benefit was achieved by the clinician making efforts to understand the participant, rather than asking the participant to make additional effort to be understood:

If I need to ramble to get things off my chest, I’ll ramble. If I’ve said a word wrong because I haven’t thought of how I needed to pronounce it, they don’t care. They won’t get me to repeat, they’ll just figure out what I meant. Whereas other clinicians would get me to repeat what I’ve just said. I don’t want to have to rethink all of that. (P1)

Suggestions for improving accommodation of autistic youth in mental health care tended to focus on options for nonverbal communication. Many participants felt they would be better able to engage with mental health care if they were able to interact in different ways, such as checking in for appointments in a way ‘that doesn’t involve any social interaction or the requirement of talking’ (P2), being able to use written communication ‘to talk about my feelings’ (P1), or expressing their need for help in a way that does not involve ‘talking face-to-face with people’ (P4). However, individual needs and preferences were variable among participants, with one highlighting the importance of clinicians documenting each client’s needs in their own words to avoid misinterpretation:

Because neurotypical people when filtering, like when writing down what someone who has ASD is saying, can tend to downplay, undersell, or completely get the wrong impression from what a sensory problem is . . . (P2).

### Theme 4. Safety planning can feel like a box-ticking exercise

Participants were specifically asked about their experiences with using suicide safety plans, as this was a standard suicide intervention utilised within the mental health services they had attended. Views were mixed, though many participants described limitations to this approach, which commonly centred around experiences of creating safety plans that felt tokenistic, generic, or ‘more for the clinician’s benefit rather than mine’ (P6).

For one participant, this sentiment came from a perception that safety plans were unrealistic for them to use during a suicidal crisis: ‘The problem that I always bring up with people for safety plans is, if I want to kill myself, why the hell am I going to read some piece of paper?’ (P2). Another reflected on how their plan was not updated often enough to remain relevant: ‘a lot of clinicians don’t update safety plans because they think they stay the same . . . Like, I know in one of my safety plans, my ex is on it. I don’t talk to my ex anymore’ (P1).

One participant felt that safety planning was approached by their clinician in a generic and tokenistic way, which undermined their ability to create a plan that truly reflected their needs: ‘I almost feel like I have to say the right thing for the therapist, rather than actually figuring out what is helpful, which is really frustrating . . .’ (P6).

Of participants who had found safety planning to be helpful, one described that their plan was a tool for reassurance whenever they felt unsafe; they had ‘looked back on it and it sort of helps you relax’ (P4). Another noted that safety planning initially felt tokenistic, but that the helpfulness of their safety plans changed over time as they developed increased awareness of the strategies and supports that were most helpful for them personally:

When I first did them, it just felt like I was doing it for the sake of doing it. Now when I do them, I know more. I guess it has been helpful when I actually know what actually does help me and not putting stuff down what I think will help me. (P3)

## Discussion

Rates of self-harm and suicidal ideation are high among autistic youth, and autistic individuals experience substantial and unique barriers to adequate mental health care (Adams & Young, 2021; O’Halloran et al., 2022). To better understand the needs of this group regarding self-harm, suicidal ideation and mental health care, we used reflexive thematic analysis to generate themes from interviews with seven autistic youth who had relevant lived experience.

Social isolation, peer rejection and bullying victimisation featured strongly as factors preceding self-harm or suicidal ideation among participants in this study, as reported in theme one. However, relationships with family members, friends and educators appeared to be important avenues of informal support, and a reason that some participants delayed self-harm or suicidal behaviour. Although these are well established risk and protective factors for self-harm and suicidal ideation among the general youth population (Arango et al., 2024; Calati et al., 2019; Cheek et al., 2020), autistic youth experience unique barriers to social connection related to social stigma regarding autism and autistic traits (Turnock et al., 2022), as well as high rates of adverse social experiences like bullying victimisation (Trundle et al., 2022), which may contribute to the elevated risk of self-harm and suicide in this population (O’Halloran et al., 2022). Satisfactory peer and parental support have been associated with lower risk of suicide among the general youth population (LeCloux et al., 2016; Scardera et al., 2020), though the role of these informal supports as potential protective factors specifically among autistic youth is underexplored in the peer-reviewed literature to-date. Indeed, there is a notable lack of investigation into broader protective factors against self-harm and suicidal ideation among autistic youth specifically, with very few exceptions (e.g. Masi et al., 2020). Future studies addressing this knowledge gap should consider the role of peer and family support.

As reported in theme two, many participants in the present study described challenges expressing or communicating about how they were feeling, with overwhelming and unexpressed emotions precipitating self-harm. Difficulties identifying the onset of a crisis point that would lead to self-harm, as well as some participants’ preferences or ability to communicate about how they were feeling, appeared to impede help-seeking at these critical times. Nearly half of autistic people experience alexithymia (i.e. difficulty identifying and describing emotions; Kinnaird et al., 2019), which may create challenges communicating about their distress or suicidal ideation, including within clinical settings (Brede et al., 2022). In addition, research suggests that clinicians are significantly less likely to identify autistic consumers as being at risk of suicide compared with non-autistic consumers (Jager-Hyman et al., 2020), creating further barriers to early identification and intervention. Although some evidence-informed resources have been developed to support mental health professionals and crisis services to better identify warning signs for self-harm and suicide in the autistic population (e.g. Hughes et al., 2020; Morgan et al., n.d.; Morgan & Maddox, 2023), the extent to which these resources have been implemented is unclear, and they do not appear to have been developed with involvement of autistic young people. There is a clear opportunity for research employing robust methodologies such as the Delphi expert consensus method (Jorm, 2016) to develop best practice clinical guidance regarding self-harm and suicidal ideation among autistic youth, including those who are non-speaking. In addition, tailored resources for parents, caregivers and other relevant adults (e.g. educators) may facilitate identification of self-harm or suicide risk among autistic youth in community settings, enabling timely intervention, especially in circumstances where the young person is unable to verbalise their distress. Wherever possible, guidelines should be co-designed with autistic young people and the autism community to optimise relevance, suitability and effectiveness.

Most participants in the present study described experiences of inadequate mental health care, largely associated with perceptions of inadequate clinician knowledge and poor accommodation of their individual needs, encapsulated in theme three. These experiences were associated with a range of negative outcomes among participants, such as distress arising from inadequate accommodations, additional burden of providing education and self-advocacy, perceiving the care as ineffective, and deterring future help-seeking. This is not dissimilar to accounts by autistic adults, who have perceived mental health professionals as lacking knowledge of autism (Brede et al., 2022; Marsden et al., 2024; Nicolaidis et al., 2015), and who have described invalidating and inadequate mental health care as a factor that contributed to, or worsened, their sense of isolation, hopelessness and distress (Camm-Crosbie et al., 2019). Adolescence is an important transitional period during which most mental health concerns emerge (Solmi et al., 2022), and evidence suggests that those who receive an autism diagnosis during adolescence are at high risk of developing depression and self-harm (Hosozawa et al., 2020). Furthermore, health-related stressors, including experiences of treatment, are significantly associated with suicidal thoughts and behaviours among autistic adults (Moseley et al., 2024). It is therefore critical that clinicians are adequately knowledgeable about autism and trained in working with autistic consumers, and that youth mental health services are equipped to provide high-quality mental health care to autistic young people, to prevent iatrogenic effects of inadequate care and to address self-harm and suicidal ideation early and effectively. In the Australian context, efforts to improve neurodiversity-affirming mental health care are evident in the Psychology Board of Australia’s recent updates to professional competencies, which will require registered psychologists to demonstrate understanding of neurodiversity and ability to reasonably accommodate people with a disability from December 2025 (see competency 7.9; Psychology Board of Australia, 2024). This update is accompanied by new continuing professional development training related to autism, which may play an important role in improving the quality of mental health care provided to autistic youth, including for self-harm and suicide; pilot data among a small sample of paediatric emergency physicians has indicated that prior training in autism, but not youth suicide, was associated with greater confidence providing suicide-related care to autistic youth (Cervantes et al., 2025). In addition, flexible and person-centred approaches to care may be of particular benefit to autistic consumers, to accommodate heterogenous needs, preferences and strengths, and to overcome stereotyped perceptions of autism within mental health settings (Brede et al., 2022).

Finally, participants’ views of safety planning indicated a common sentiment that the intervention did not always reflect their needs and was not necessarily perceived as being for their benefit, as reported in theme four. Prior research suggests that autistic individuals may need more time than is currently provided to reflect on their needs and supports during safety planning, prompting the development of an Autism Adapted Safety Plan (AASP; Goodwin et al., 2025), which was co-produced with autistic adults and their caregivers. Novel features of AASP such as flexibility in timing of delivery (i.e. use of multiple sessions) and use of visual aids to assist with identifying and expressing emotions may address some of the limitations described by participants in the present study. Although AASP has not yet been widely adopted in clinical practice, preliminary evaluation supports its efficacy and feasibility in reducing the incidence of self-harm and suicide risk among autistic adults (Rodgers et al., 2024). Future research should evaluate AASP among autistic youth, to determine whether the effects observed in adult samples apply to younger age ranges, or whether further adaptations are required to improve the efficacy of this intervention in preventing self-harm and suicide among autistic youth.

### Strengths and limitations

This study contributes to the growing literature regarding autism, self-harm and suicide, and notably, addresses the scarcity of research exploring the experiences of autistic youth receiving mental health care for self-harm and suicidal ideation. Our recruitment approach enabled collection of data that was highly specific and relevant to the study aims, yielding sufficient information power despite the small sample size. Information power is an approach for assessing qualitative research, which posits that fewer participants are needed to achieve high-quality findings if they hold a greater amount of information relevant to the research questions (Malterud et al., 2016).

Although qualitative research is not concerned with producing generalisable results, interpretations of the present findings should consider the characteristics of the sample. As recruitment occurred through health systems situated within metropolitan Melbourne, findings may not reflect autistic young people’s experiences of mental health care in regional settings. More than half of the sample identified as transgender or gender diverse, and the remainder identified as female, meaning that participants’ experiences may be contextualised by factors such as gender identity-based discrimination or gender stereotypes regarding autism, which were not discussed here. Participants were able to communicate verbally during the interview and none reported experiencing a co-occurring intellectual disability, meaning that the possibly unique experiences of autistic youth with intellectual disability or without verbal communication ability are not reflected in the present findings. Future investigation into self-harm, suicidal ideation and mental health care among autistic youth with intellectual disability is necessary to ensure specific risk factors and health care needs relevant to this population are understood and addressed.

## Conclusion

Mental health care settings are key environments for identifying and intervening against youth self-harm and suicidal ideation. However, the findings from this study indicate that autistic youth experience particular challenges in receiving appropriate support for self-harm and suicidal ideation, due to difficulties identifying and communicating about their distress, poor knowledge and accommodation of autism among clinicians, and delivery of standard suicide prevention interventions that may not reflect the individual’s needs. These findings have been used to discuss opportunities to improve the quality of mental health care provided to autistic youth experiencing self-harm or suicidal ideation, including development of new guidelines to improve provider knowledge about self-harm and suicide in autistic youth, and utilising flexible, person-centred approaches that accommodate autistic consumers’ individual needs.

## Supplemental Material

sj-docx-1-aut-10.1177_13623613251366863 – Supplemental material for Experiences of self-harm, suicidal ideation and mental health care among autistic youth Supplemental material, sj-docx-1-aut-10.1177_13623613251366863 for Experiences of self-harm, suicidal ideation and mental health care among autistic youth by Amanda Sabo, Jo Robinson, India Bellairs-Walsh, Linda Taimre, William Nguyen, Lisa Phillips, Michelle Lamblin and Eleanor Bailey in Autism
