# Supplementary material for: Experiences of self-harm, suicidal ideation and mental health care among autistic youth
Source: Autism. 2025 Sep 1;30(1):74–83. doi: 10.1177/13623613251366863 (PMC12717283; doi:10.1177/13623613251366863)
Supplement: sj-docx-1-aut-10.1177_13623613251366863 – Supplemental material for Experiences of self-harm, suicidal ideation and mental health care among autistic youth [file sj-docx-1-aut-10.1177_13623613251366863.docx]

**Supplemental Material for:**

Experiences of self-harm, suicidal ideation and mental health care among autistic youth

**Appendix 1. Interview schedule**

| **TOPIC** | **STEM QUESTION** | **FURTHER PROMPTS** |
| --- | --- | --- |
| Initial demographics | How old are you?  How do you define your gender?  How do you define your sexuality?  What state or territory do you live in?  Are you of Aboriginal or Torres Strait Islander background?  What is your highest level of education?  Are you currently studying and/or working?  What age were you when you were diagnosed with ASD/were told you might have ASD? | (e.g. male, female, trans male, trans female, non-binary, genderqueer, a gender or another term)  (e.g. gay, lesbian, bisexual, pansexual, queer, asexual or another term) |
| Establishing the picture | Can you tell us a bit about your experience of autism and seeking help? |  |
| Strengths | What are some of your personal strengths or things that you are good at that you take pride in? | Would you find it useful for someone, like a clinician, to help you identify your strengths and abilities? |
| Gender | ASD can look different in youth of different genders.  Do you think that your gender has impacted on your experience of seeking help?*(prompt: if yes, how so?)* | For example, some youth with ASD may be better or worse at hiding or masking some of their feelings/behaviours.  Do you think being X (female, male, trans, etc.) made the experience of seeking help different for you than it would have been if you were another gender?  Did it make no difference, some difference, or a big difference to the support you received? *(prompt: if it made some/big difference – could you describe those differences?)* |
| Self-harm  Suicidal feelings | I’m now going to be asking you some questions about your experience of suicidal thoughts/self-harm. If you find this difficult, please let me know and we can stop or take a break.  As you know this study is about suicide and self-harm prevention. Can you tell us about your experiences with suicidal feelings or self-harm?  *Alternative language: wanting to hurt yourself, wishing you were dead, wanting to die, wanting to end your life* | At that time what were your thoughts/ feelings?  When you hurt yourself, were you thinking about suicide?  Thinking about the time leading up to this, what was going on for you? (thoughts/ triggers) *(prompt: What were you thinking? What were you feeling?)*  Did you think about hurting yourself *(if applicable: or wanting to die)* a lot before acting on it? *(prompt: Did you think about it once, a few times, or lots of times?)*  Have there been times you felt this way but didn’t act – what stopped you? |
| Seeking help for self-harm and/or suicidal feelings specifically | When you hurt yourself or wanted to die, did you get help from a mental health service? Can you tell us a bit about this?  What worked well for you?  What didn’t work well for you?  What other strategies could clinicians/services use that would be helpful? (e.g. using special sensory equipment, writing down what you wanted to say, sensory toys etc.)  What could mental health services do differently in the future?  (Check in and ask if they are ok to keep going at this point) | Do you think clinicians were sufficiently equipped to meet your needs?  Did they have the skills and time to help you?  When talking to clinicians, do you feel like you can be yourself? Do you feel the clinicians accept you as you are?  *Alternative language: your full self, express yourself freely, have to hide parts of who you are, have to change how you talk or act*  Did anything make it easier to get the support you needed from the mental health service?  Did you feel like the service/clinicians considered any sensory or processing issues that you may have?  *Alternative language: how people react to sound or light, how people think, how fast or slow people talk* |
| Safety planning | When someone wants to hurt themselves or wants to die, clinicians often work with that person to make a safety plan, to help them get support and stay safe. Have you ever used a safety plan?  *Alternative language: a plan that identifies coping and help-seeking strategies that are tailored for their needs, situation, and personal relationships; plans that identify ways that help the person stay safe, which could ways to get them help that are designed especially for them* | If yes, what was your experience with a safety plan?  If no, is that something that you can imagine being helpful?  Are there people you feel comfortable reaching out to in times of crisis?  *Alternative language: friends, groups of friends, social clubs, social groups, healthcare worker*  In times of crisis, what would help to best communicate your need for support? |
| COVID-19 | Given we are doing this interview and asking you to think about all this in the midst of a pandemic, can I ask what impact (if any) do you think the current coronavirus pandemic has had on you personally and your mental health?  Has the pandemic affected how you would usually see your clinician? (e.g. over zoom/phone)? How are you finding this? |  |
| Check in/Mood elevating activity e.g. mindfulness exercise | This is all the questions we have.  We’ve talked about a lot of things today, I want to check is there anything else you would like to add about the care you have had or would like to receive in the future?  And can I ask if you feel ok after all that?  We often find that it’s nice to finish on a happier/ more relaxing note. Some people like to do a short mindfulness exercise, or watch a relaxing nature video, or maybe listen to their favourite song. Would you like to do something like that together before we finish up? | Before the interview you told us you used X or Y as coping strategies, let’s discuss how you know your signs of distress and what coping strategies you have found to be effective?  What are you doing this weekend? Are you looking forward to…? |
